# Goal-directed therapy in cancer surgery: a randomised and controlled trial (GRICS II)

**DOI:** 10.1186/2197-425X-3-S1-A819

**Published:** 2015-10-01

**Authors:** A Gerent, JP Almeida, F Galas, JT Fukushima, E Osawa, C Park, R Franco, Y Sakr, LA Hajjar

**Affiliations:** Instituto do Câncer do Estado de São Paulo, Anesthesia and Critical Care, São Paulo, Brazil; Friedrich-Schiller University, Jena, Germany

## Introduction

A perioperative hemodynamic goal-directed therapy (GDT) has been advocated in patients undergoing high risk-surgery in order to reduce postoperative complications. Nevertheless, the benefits of GDT in surgical patients are controversial and may lead to an overuse of fluids, inotropic agents and blood transfusion.

## Objectives

The aim of this randomized, controlled, single-center trial was to assess the effect of GDT after abdominal oncology surgery on the incidence of major postoperative complications. We also evaluated the requirements of fluid, inotropic agents, blood transfusion and the intensive care unit (ICU) and hospital length of stay between groups.

## Methods

We randomly assigned patients admitted to the ICU after major abdominal oncology surgery to receive either GDT protocol or usual care. The GDT protocol involved hemodynamic optimization aimed at a target of a cardiac index >2.5 L/minute/m2 (by minimally invasive cardiac output monitoring) and a mean arterial pressure of 70 mmHg through a three-step approach: fluid therapy of 250 ml albumin 4% in lactated *Ringer´s* solution, dobutamine infusion up to a dose of 20 µg/kg/minute, and red blood cell transfusion to reach a hemoglobin level above 8g/dL. The primary outcome was a 30-day composite endpoint of acute kidney injury, major cardiovascular complications, adult respiratory distress syndrome, septic shock, reoperation and mortality.

## Results

Of the 125 enrolled patients, 62 were assigned to GDT-group and 63 to the usual-care group. There was no difference between groups regarding the amount of fluids received during 8 hours (1345 mL [1042-1771] vs. 1100 mL [780-1388], p = 0.151) of intervention and during 24 hours (2021 mL [1527-2844] vs. 2010 mL [1747-2410], p = 0.372). Patients in the GDT-group were more likely to receive dobutamine (37[66.1%] vs. 12 [23.1%],p < 0.0001). At 30 days after randomization, the composite endpoint occurred in 25.8% in the GDT group and in 22.2% in the usual-care group (p = 0.639). There was no significant difference in blood transfusion requirements, in-hospital mortality, duration of organ support, or length of hospital stay.

## Conclusions

In surgical critically ill patients admitted to the ICU after major abdominal oncology surgery, a GDT protocol did not reduce the incidence of major postoperative complications at 30 days. Also, the GDT protocol was associated with an overuse of inotropic agents. (ClinicalTrials.gov number, NCT 01946269.)

## Conflict of interest

No conflict of interest to disclose
